# Supplementary material for: Swap Up Your Meal: A Mass Media Nutrition Education Campaign for Oklahoma Teens
Source: Int J Environ Res Public Health. 2022 Aug 16;19(16):10110. doi: 10.3390/ijerph191610110 (PMC9408208; doi:10.3390/ijerph191610110)
Supplement: Supplementary file 1 [file ijerph-19-10110-s001.zip › ijerph-1813882-supplementary.pdf]

International Journal of
Environmental Research and Public Health

MDPI

Table S1: *Swap Up* Main Message Awareness Items and Frequencies

Item	Total % (*n* = 200)	Urban % (*n*)	Rural % (*n*)	Overweight % (*n*)	Not Overweight % (*n*)	Aware % (*n*)	Not Aware % (*n*)	Engaged % (*n*)	Not Engaged % (*n*)
Overall	83% (166)	59% (117)	25% (49)	48% (95)	36% (71)	66% (131)*	18% (35)	41% (82)	42% (84)*
Eat how you want to feel	35% (70)	24% (48)	11% (22)	15% (29)	21% (41)	27% (53)	9% (17)	19% (38)*	16% (32)
Healthy foods power you up	34% (68)	25% (49)	10% (19)	15% (29)	20% (39)	29% (58)*	5% (10)	19% (37)*	16% (31)
Unhealthy foods drag you down	43% (85)	29% (57)	14% (28)	19% (37)	24% (48)	38% (75)	5% (10)	24% (48)*	19% (37)
Sugar holds you back	39% (77)	26% (52)	13% (25)	16% (32)	23% (45)	35% (69)*	4% (8)	23% (46)*	16% (31)
Start swapping up	26% (51)	19% (38)	7% (13)	13% (26)	13% (25)	21% (41)	5% (10)	17% (33)*	9% (18)
Greasy foods slow you down	43% (86)	30% (60)	13% (26)	17% (34)	26% (52)	36% (72)*	7% (14)	23% (45)*	21% (41)
Sugar causes crashes	42% (83)	27% (53)	15% (30)	19% (38)	23%(45)	37% (73)*	5% (10)	23% (45)*	19% (38)
Fruits and veggies give you energy	45% (89)	36% (71)*	9% (18)	20% (40)	25% (49)	34% (68)	11% (21)	21% (42)	24% (47)
Water refreshes you	46% (91)	32% (64)	14% (27)	22% (44)	24% (47)	36% (71)	10% (20)	24% (48)*	22% (43)
Water energizes you	42% (84)	28% (56)	14% (28)	21% (41)	22% (43)	34% (68)*	8% (16)	18% (38)	24% (48)

*Significantly different at the $p < 0.05$ level.

Table S2: Increasing Water Items and Frequencies.

Item	Total % (n = 200)	Urban % (n)	Rural % (n)	Overweight % (n)	Not Overweight % (n)	Aware % (n)	Not Aware % (n)	Engaged % (n)	Not Engaged % (n)
Overall	78% (156)	55% (109)	24% (47)	35% (69)	44% (87)	59% (118)*	19% (38)	38% (75)	41% (81)*
Set a goal to drink more water	62% (103)	46% (76)	16% (27)	25% (41)	38% (62)	48% (79)	15% (24)	30% (50)	32% (53)
Told my friends about my plans to drink more water	17% (28)	13% (21)	4% (7)	10% (17)	7% (11)	13% (22)	4% (6)	12% (20)*	5% (8)
Told my family about my plans to drink more water	25% (41)	16% (27)	9% (14)	12% (19)	13% (22)	19% (32)	5% (9)	14% (23)	11% (18)
Looked up information (e.g. online) about tips for drinking more water	16% (27)	13% (22)	3% (5)	8% (13)	9% (14)	11% (18)	5% (9)	11% (18)*	5% (9)
Looked up information about how drinking water can make you think, act	15% (25)	13% (22)*	2% (3)	8% (13)	7% (12)	12% (19)	4% (6)	11% (18)*	4% (7)

or feel better									
Looked up ways to make water taste more interesting (e.g. infused water recipes)	24% (*39*)	19% (*31*)	5% (*8*)	13% (*21*)	11% (*18*)	21% (*35*)*	2% (*4*)	17% (*28*)*	7% (*11*)
Purchased, or asked others to purchase for me sparkling unsweetened water or bottled water	35% (*58*)	26% (*43*)	9% (*15*)	15% (*25*)	20% (*33*)	28% (*47*)	7% (*11*)	16% (*27*)	19% (*31*)
Purchased, or asked others to purchase for me a refillable water bottle or a water filter	33% (*55*)	22% (*37*)	11% (*18*)	13% (*21*)	21% (*34*)	28% (*46*)	5% (*9*)	19% (*31*)	15% (*24*)
Asked a friend to help in some way	10% (*16*)	6% (*9*)	4% (*7*)	4% (*7*)	6% (*9*)	8% (*13*)	2% (*3*)	7% (*11*)	3% (*5*)
Downloaded or looked into an app that would let me track my water	20% (*33*)	15% (*25*)	5% (*8*)	10% (*17*)	10% (*16*)	17% (*28*)	3% (*5*)	13% (*21*)*	7% (*12*)
Began tracking how much water I drink (e.g. through a chart, app)	28% (*46*)	19% (*32*)	9% (*14*)	13% (*22*)	15% (*24*)	22% (*36*)	6% (*10*)	15% (*25*)	13% (*21*)

*Significantly different at the $p < 0.05$ level.

Table S3: Decreasing Sugary Drinks Items and Frequencies.

Item	Total % (*n* = 200)	Urban % (*n*)	Rural % (*n*)	Overweight % (*n*)	Not Overweight % (*n*)	Aware % (*n*)	Not Aware % (*n*)	Engaged % (*n*)	Not Engaged % (*n*)
Overall	60% (*119*)	45% (*89*)	15% (*30*)	25% (*50*)	35% (*69*)	46% (*91*)	14% (*28*)	32% (*63*)*	28% (*56*)
Told my friends about my plans to drink fewer sodas/sugary drinks	23% (*30*)	16% (*21*)	7% (*9*)	11% (*15*)	11% (*15*)	19% (*25*)	4% (*5*)	14% (*18*)	9% (*12*)
Told my family about my plans to drink fewer sodas/sugary drinks	24% (*31*)	18% (*24*)	5% (*7*)	11% (*15*)	12% (*16*)	19% (*25*)	5% (*6*)	17% (*22*)*	7% (*9*)
Looked up information (e.g. online) about tips for drinking fewer sodas/sugary drinks	15% (*20*)	14% (*19*)*	1% (*1*)	7% (*9*)	8% (*11*)	14% (*18*)	2% (*2*)	10% (*13*)	5% (*7*)
Looked up information about how drinking sodas/sugary drinks can make you think, act or feel worse	15% (*20*)	13% (*17*)	2% (*3*)	6% (*8*)	9% (*12*)*	15% (*20*)	0% (*0*)	9% (*12*)	6% (*8*)
Asked my parents to stop buying these drinks	27% (*36*)	21% (*27*)	7% (*9*)	12% (*16*)	15% (*20*)	18% (*24*)	9% (*12*)	16% (*21*)	11% (*15*)
Got rid of some of these drinks that I had at my house	22% (*29*)	17% (*23*)	5% (*6*)	10% (*13*)	12% (*16*)	18% (*24*)	4% (*5*)	13% (*17*)	9% (*12*)

Moved some of these drinks so they were harder to access (e.g. put on a high shelf)	17% (22)	13% (17)	4% (5)	9% (12)	8% (10)	13% (17)	4% (5)	11% (15)	5% (7)
Avoided people, places or activities that would make me want to drink sodas/sugary drinks	32% (42)	25% (33)	7% (9)	12% (16)	20% (26)	24% (32)	8% (10)	17% (23)	14% (19)
Asked a friend to help in some way	10% (13)	6% (8)	4% (5)	6% (8)	4% (5)	8% (11)	2% (2)	8% (11)*	2% (2)
Purchased or had someone purchase for me a "diet" beverage instead of a "regular" soda/sugary beverage	29% (38)	24% (31)	5% (7)	13% (17)	16% (21)	22% (29)	7% (9)	14% (18)	15% (20)
Downloaded or began using an app that lets me track my sodas/sugary drinks	12% (16)	9% (12)	3% (4)	8% (10)	5% (6)	11% (14)	2% (2)	8% (11)	4% (5)
Set a limit on how many sodas/sugary drinks I should drink each day, or limited the times/days I drink them	45% (59)	34% (45)	11% (14)	17% (22)	28% (37)	33% (44)	11% (15)	23% (31)	21% (28)
Began tracking how many sodas/sugary drinks I drink (e.g. through a chart or app)	24% (31)	18% (24)	5% (7)	11% (14)	13% (17)	20% (26)	4% (5)	12% (16)	11% (15)
While I still had these drinks, I tried to drink a bit less of them by taking a smaller serving or not finishing all of the drink	36% (47)	29% (38)	7% (9)	19% (25)	17% (22)	26% (34)	10% (13)	20% (27)	15% (20)

*Significantly different at the $p < 0.05$ level.

Table S4: Increasing Fruits and Veggies Items and Frequencies.

Item	Total % (n = 200)	Urban % (n)	Rural % (n)	Overweight % (n)	Not Overweight % (n)	Aware % (n)	Not Aware % (n)	Engaged % (n)	Not Engaged % (n)
Overall	67% (134)	48% (96)	19% (38)	30% (60)	37% (74)	52% (104)*	15% (30)	34% (67)	34% (67)
Set a goal to eat more fruits/vegetables	44% (65)	32% (47)	12% (18)	19% (28)	25% (37)	34% (51)	9% (14)	23% (34)	21% (31)
Told my friends about my plans to eat more fruits/vegetables	21% (31)	16% (24)	5% (7)	10% (15)	11% (16)	17% (25)	4% (6)	13% (19)	8% (12)
Told my family about my plans to eat more fruits/vegetables	35% (51)	26% (39)	8% (12)	16% (24)	18% (27)	27% (40)	7% (11)	18% (26)	17% (25)
Looked up information (e.g. online) about tips for eating more fruits/vegetables	31% (46)	23% (34)	8% (12)	16% (23)	16% (23)	27% (40)	4% (6)	21% (31)*	10% (15)
Looked up recipes or information about how to prepare fruits/vegetables	35% (51)	24% (36)	10% (15)	15% (22)	20% (29)	27% (40)	7% (11)	20% (29)	15% (22)

Looked up information about how eating fruits/vegetables can make you think, act or feel better	21% (*31*)	16% (*23*)	5% (*8*)	12% (*17*)	10% (*14*)	17% (*25*)	4% (*6*)	14% (*20*)*	7% (*11*)
Purchased, or asked others to purchase, more fruits/vegetables to have at home	61% (*90*)	42% (*62*)	19% (*28*)	29% (*43*)	32% (*47*)	46% (*68*)	15% (*22*)	28% (*42*)	32% (*48*)
Put fruits/vegetables in a place where I would see them more easily	34% (*50*)	27% (*40*)	7% (*10*)	14% (*20*)	20% (*30*)	26% (*38*)	8% (*12*)	16% (*23*)	18% (*27*)
Tried a type of fruit/vegetable I typically do not eat	28% (*42*)	20% (*30*)	8% (*12*)	14% (*20*)	15% (*22*)	20% (*30*)	8% (*12*)	15% (*22*)	14% (*20*)
Asked a friend to help in some way	14% (*21*)	12% (*17*)	3% (*4*)	7% (*10*)	7% (*11*)	11% (*17*)	3% (*4*)	11% (*16*)*	3% (*5*)
Downloaded or looked into an app related to nutrition	22% (*32*)	18% (*26*)	4% (*6*)	12% (*18*)	10% (*14*)	17% (*25*)	5% (*7*)	14% (*20*)	8% (*12*)
Began tracking how much fruits/vegetables I eat (e.g. through a chart, app)	24% (*35*)	18% (*26*)	6% (*9*)	10% (*15*)	14% (*20*)	20% (*30*)	3% (*5*)	14% (*21*)	9% (*14*)

*Significantly different at the $p < 0.05$ level.

Table S5: Decreasing Greasy, Fried, and Sugary Foods Items and Frequencies.

Item	Total % (n = 200)	Urban % (n)	Rural % (n)	Overweight % (n)	Not Overweight % (n)	Aware % (n)	Not Aware % (n)	Engaged % (n)	Not Engaged % (n)
Overall	65% (*129*)	46% (*91*)	19% (*38*)	28% (*56*)	37% (*73*)	48% (*96*)	17% (*33*)	30% (*60*)	35% (*69*)
Set a goal to eat less of these foods	48% (*67*)	37% (*51*)	12% (*16*)	20% (*28*)	28% (*39*)	37% (*51*)	12% (*16*)	23% (*32*)	25% (*35*)
Told my friends about my plans to eat less of these foods	20% (*28*)	14% (*20*)	6% (*8*)	11% (*15*)	9% (*13*)	15% (*21*)	5% (*7*)	12% (*16*)	9% (*12*)
Told my family about my plans to eat less of these foods	25% (*34*)	19% (*26*)	6% (*8*)	12% (*16*)	13% (*18*)	19% (*26*)	6% (*8*)	10% (*14*)	14% (*20*)
Looked up information (e.g. online) about tips for eating less of these foods	19% (*27*)	17% (*23*)*	3% (*4*)	9% (*13*)	10% (*14*)	15% (*21*)	4% (*6*)	14% (*19*)*	6% (*8*)
Looked up information about how these foods can make you think, act or feel worse	18% (*25*)	12% (*17*)	6% (*8*)	8% (*11*)	10% (*14*)	14% (*20*)	4% (*5*)	13% (*18*)*	5% (*7*)
Asked my parents to stop buying these foods	24% (*33*)	19% (*26*)	5% (*7*)	10% (*14*)	14% (*19*)	19% (*26*)	5% (*7*)	14% (*19*)	10% (*14*)
Got rid of some of these foods that I had at my house	28% (*39*)	19% (*26*)	9% (*13*)	14% (*20*)	14% (*19*)	22% (*31*)	6% (*8*)	19% (*26*)*	9% (*13*)
Moved some of these foods so they were	24% (*33*)	21% (*29*)*	3% (*4*)	8% (*11*)	16% (*22*)	18% (*25*)	6% (*8*)	17% (*23*)*	7% (*10*)

harder to access (e.g. put on a high shelf)									
Tried to find different ways to relax or manage stress/boredom	40% (*56*)	27% (*38*)	13% (*18*)	18% (*25*)	22% (*31*)	31% (*43*)	9% (*13*)	20% (*28*)	20% (*28*)
Avoided people, places or activities that would make me want to eat these foods	32% (*44*)	25% (*34*)	7% (*10*)	14% (*20*)	17% (*24*)	14% (*33*)	8% (*11*)	15% (*21*)	17% (*23*)
Asked a friend to help in some way	8% (*11*)	7% (*9*)	1% (*2*)	4% (*6*)	4% (*5*)	6% (*9*)	1% (*2*)	5% (*7*)	3% (*4*)
Downloaded or began using an app related to nutrition	21% (*29*)	16% (*22*)	5% (*7*)	12% (*17*)	9% (*12*)	13% (*18*)	8% (*11*)	10% (*14*)	11% (*15*)
Began tracking how much of these foods I eat (e.g. through a chart or app)	23% (*32*)	17% (*24*)	6% (*8*)	11% (*15*)	12% (*17*)	17% (*23*)	6% (*9*)	14% (*20*)	9% (*12*)
While I still had these foods, I tried to eat a bit less of them by taking a smaller serving or not finishing all of the food	53% (*73*)	35% (*49*)	17% (*24*)	27% (*37*)	26% (*36*)	37% (*51*)	16% (*22*)	27% (*37*)	26% (*36*)

*Significantly different at the $p < 0.05$ level.

Table S6: *Swap Up* Digital Engagement Items and Frequencies.

Item	**Total % (n = 200)**	**Urban % (n)**	**Rural % (n)**	**Overweight % (n)**	**Not Overweight % (n)**	**Aware % (n)**	**Not Aware % (n)**	**Engaged % (n)**	**Not Engaged % (n)**
Overall	44% *(87)*	30% *(60)*	14% *(27)*	21% *(41)*	23% *(46)*	38% *(75)* *	6% *(12)*	-	-
Seen a GIF/short video from *Swap Up* online	22% (*44*)	14% (*27*)	9% (*17*)	12% (*24*)	10% (*20*)	19% (*37*)*	4% (*7*)	22% (*44*)*	0% (*0*)
Opened or seen a story on social media from *Swap Up*	22% (*44*)	15% (*29*)	8% (*15*)	10% (*20*)	12% (*24*)	20% (*40*)*	2% (*4*)	22% (*44*)*	0% (*0*)
Liked, reacted or saved a post from *Swap Up* on social media	9% (*17*)	7% (*13*)	2% (*4*)	5% (*9*)	4% (*8*)	7% (*14*)	2% (*3*)	9% (*17*)*	0% (*0*)
Swiped up on a *Swap Up* ad or clicked a link or button on social media	8% (*16*)	7% (*13*)	2% (*3*)	4% (*7*)	5% (*9*)	7% (*13*)	2% (*3*)	8% (*16*)*	0% (*0*)
Commented or shared content from *Swap Up* on social media	5% (*9*)	4% (*7*)	1% (*2*)	3% (*6*)	2% (*3*)	4% (*8*)	1% (*1*)	5% (*9*)*	0% (*0*)

*Significantly different at the $p < 0.05$ level.
